# Temporal Trends in Mortality Related to Hyperthyroidism and Heart Failure in the United States

**DOI:** 10.7759/cureus.86274

**Published:** 2025-06-18

**Authors:** Amruth A Alluri, Namita Salahuddin, Tishya Mujoo, Soukhya Neelam, Nitish Thirugnanasambandam, Priyank Shah

**Affiliations:** 1 Internal Medicine, American University of the Caribbean School of Medicine, Cupecoy, SXM; 2 Internal Medicine, Essen Health Care, New York, USA; 3 Cardiology, Ravindra Nath Tagore Medical College, Udaipur, Udaipur, IND; 4 Internal Medicine, Government Medical College, Nagarkurnool, Nagarkurnool, IND; 5 Internal Medicine, Dr. RK Diabetic Foot and Podiatry Institute, Chennai, IND; 6 Internal Medicine, Shiv Hospital, Patdi, IND

**Keywords:** age-adjusted mortality rate, cdc mcd, heart failure, hyperthyroidism, retrospective study

## Abstract

Introduction: Hyperthyroidism is one of the most frequently diagnosed disorders in the United States (US), and its association with heart failure (HF) remains underexplored. Understanding this relationship is critical for identifying high-risk populations and guiding preventive efforts.

Aims: This study aimed to analyze temporal and demographic trends in mortality where hyperthyroidism was the underlying cause and HF a contributing cause, using national data from 1999 to 2020.

Methodology: A retrospective observational study was conducted using the Centers for Disease Control and Prevention (CDC) Multiple Causes of Death (MCD) database to assess mortality trends in individuals aged ≥25 years in the US from 1999 to 2020. The study included deaths in which hyperthyroidism (ICD-E05) was listed as an underlying cause and HF (ICD-i50) as a contributing cause. Data were analyzed by gender, race, geographic area, and place of death. Age-adjusted mortality rates (AAMRs) and annual percentage changes (APCs) were calculated.

Results: A total of 1,189 deaths were documented. The AAMR initially increased (25.95% APC from 1999 to 2001), followed by a decline from 2001 to 2011 (-5.04% APC); however, it increased from 2011 to 2020 (1.83% APC). The highest mortality was observed in females (70%), White individuals (73.93%), and those living in metropolitan regions (75.4%). Temporal trends showed an increasing AAMR in females (APC: +3.74% after 2013) and White individuals (APC: 8.16% from 2017 to 2020), indicating evolving disparities.

Conclusions: Mortality trends in hyperthyroidism with HF have shifted, with increasing disparities in gender and race. These findings emphasize the need for targeted prevention strategies and improved healthcare access.

## Introduction

Hyperthyroidism is a common endocrine disorder in the United States (US) with a prevalence of 1.2% [1]. It involves the overproduction and release of thyroid hormones from the thyroid gland. Hyperthyroidism is more prevalent in women than in men (2% vs. 0.2%, respectively). The National Health and Nutrition Examination Survey (NHANES) data show that thyrotoxicosis is nearly three times more common in non-Hispanic Black individuals than in White individuals. This variation may be due to genetic predisposition, environmental influences, and differences in healthcare access [2,3]. Thyroid storm is the most critical and fatal manifestation of hyperthyroidism, with a mortality rate of 8-25% [4,5].

Heart failure (HF) is the inability of the heart to pump sufficient blood to meet the body's metabolic demands. As of 2024, approximately 6.7 million Americans aged >20 years have HF, which is projected to rise to 8.7 million by 2030 [6]. The Framingham Heart Study reported an HF incidence of 0.3% per year in men and 0.2% in women (50-59 years), increasing to 2.7% and 2.2%, respectively, at 80-89 years, with men having a 1.75 times higher risk at all ages [7]. The age-standardized HF incidence decreased from 3.9% in 2000 to 3.0% in 2017 in lagging states and from 2.9% to 2.2% in leading states [8]. Black men and women have higher cardiovascular mortality rates than White individuals [9].

The heart is sensitive to changes in thyroid hormone concentrations because of the presence of thyroid hormone receptors. Thyroid dysfunction can lead to endothelial dysfunction, blood pressure changes, myocardial dysfunction, and dyslipidemia [10]. Hyperthyroidism can worsen cardiac conditions by increasing the myocardial oxygen demand, contractility, and heart rate [11]. Udani (2021) found that patients with HF and thyroid dysfunction had a 60% greater mortality risk than those with normal thyroid function [12]. Fan (2024) indicated that approximately 8% of patients with hyperthyroidism develop HF, with atrial fibrillation being the most common risk factor [13]. Research on HF mortality often overlooks the impact of hyperthyroidism, especially in subclinical cases and in different demographic groups. Only a few studies have examined the influence of hyperthyroidism on HF-related mortality at the population level in the elderly. Using the Centers for Disease Control and Prevention (CDC) Multiple Causes of Death (MCD) database, national mortality trends can be uncovered, subclinical impacts assessed, and risk stratification refined, leading to improved patient management and more targeted healthcare interventions.

This study aimed to analyze mortality trends in hyperthyroidism with HF as a contributing cause of death using the CDC MCD database and explore trends from 1999 to 2020, analyzing data by sex, race, and geographic location to identify disparities in mortality patterns.

## Materials and methods

A retrospective original research study was conducted using the Center for Disease Control and Prevention Wide-Ranging Online Data for Epidemiologic Research (CDC WONDER) MCD database [14]. This study utilized publicly available mortality data, including de-identified death certificate information for all deaths recorded in the US. Data extraction will be performed on January 20, 2025. As the dataset consisted of publicly available, de-identified information, the study was classified as non-human participant research and was thus exempt from Institutional Review Board (IRB) approval [15].

Mortality data were extracted from the CDC WONDER MCD database for the years 1999-2020. The study included individuals aged ≥25 years, as hyperthyroidism-related mortality is rare in younger populations. Hyperthyroidism (ICD-10: E05) was selected as the underlying cause of death, and HF (ICD-10: I50) was selected as the multiple cause of death to assess the co-occurrence of these conditions. Demographic variables such as sex (male and female) and race/ethnicity (American Indian or Alaska Native, Asian or Pacific Islander, Black or African American, and White) were included to analyze disparities in mortality outcomes. Geographic variables included urbanization based on the 2013 classification, categorizing metropolitan cities into large central metro, large fringe metro, medium metro, and small metro, and non-metropolitan cities into micropolitan and non-core rural areas. Additionally, the place of death was categorized as a medical facility, decedent's home, hospice facility, nursing home/long-term care facility, or other. Mortality rates were standardized using age-adjusted rates per 1,000,000 population, with adjustments based on the US Standard Population from 2000 to allow accurate comparisons over time.

Descriptive statistics, including absolute numbers and percentages, were used to summarize the demographic and geographic variables. Age-adjusted mortality rates (AAMRs) were calculated for each subgroup using the CDC WONDER MCD database. Joinpoint regression analysis (version 5.3.0.0, November 2024) was used to identify statistically significant changes in mortality trends, with joinpoints added where there were sudden or directional shifts in AAMRs. The maximum number of joinpoints was selected using the Bayesian Information Criterion (BIC), and model selection was guided by permutation tests. Subgroup trends with <10 deaths were suppressed due to CDC privacy rules, and missing denominators limited certain subgroup analyses. Trends were assessed over the 1999-2020 study period to identify statistically significant changes in mortality patterns across different demographic and geographic groups.

## Results

During the study period from 1999 to 2020, the CDC MCD database recorded 1189 deaths in the US in individuals aged >25 years. The results included deaths in which hyperthyroidism (ICD-E05) was listed as the underlying cause of death and HF (ICD-I50) was noted as a contributing cause of death (1189). The crude mortality rate for hyperthyroidism with HF as a contributing cause was 0.3 per 1,000,000 individuals. Deaths due to causes other than the criteria were excluded from the study.

Demographic characteristics

From the study, males accounted for 357 (30%), while females accounted for 832 (70%) of the total deaths (Table 1). Compared to males in the study population, the mortality rate for hyperthyroidism with HF as a contributing cause was higher in females. This indicates a potential demographic disparity in these results. Regarding racial distribution, the highest proportion of deaths occurred among White individuals (n = 879, 73.93%), followed by Black or African American (n = 267, 22.46%) and Asian or Pacific Islander individuals (n = 36, 3.03%). The mortality burden was highest among White individuals, highlighting racial disparities in mortality trends related to hyperthyroidism and HF.

**Table 1:** Distribution of deaths by urbanization level, place of death, gender, and race

Urbanization	Number of deaths	Percentage
Metropolitan area	897	75.4
Large Central Metro	327	27.5
Large Fringe Metro	216	18.2
Medium Metro	230	19.3
Small Metro	124	10.4
Non-metropolitan area	292	24.5
Micropolitan	150	12.6
Non-core	142	11.9
Place of death	Number of deaths	Percentage
Medical facility	651	54.7
Decedent's home	256	21.5
Hospice facility	18	1.5
Nursing home/long-term care	225	18.9
Other	32	2.7
Gender	Number of deaths	Percentage
Male	357	30
Female	832	70
Race	Number of deaths	Percentage
Asian or Pacific Islander	36	3.03
Black or African American	267	22.46
White	879	73.93

Geographic characteristics

The majority of deaths occurred in metropolitan areas (n = 897, 75.4%), while non-metropolitan areas accounted for n = 292, 24.5% of deaths. Regarding the place of death, most deaths occurred in medical facilities (n = 651, 54.7%), followed by dependent homes (n = 256, 21.5%), nursing homes/long-term care (n = 225, 18.9%), and hospice facilities (n = 18, 1.5%) (Table 1).

Temporal trends

**Figure 1:**
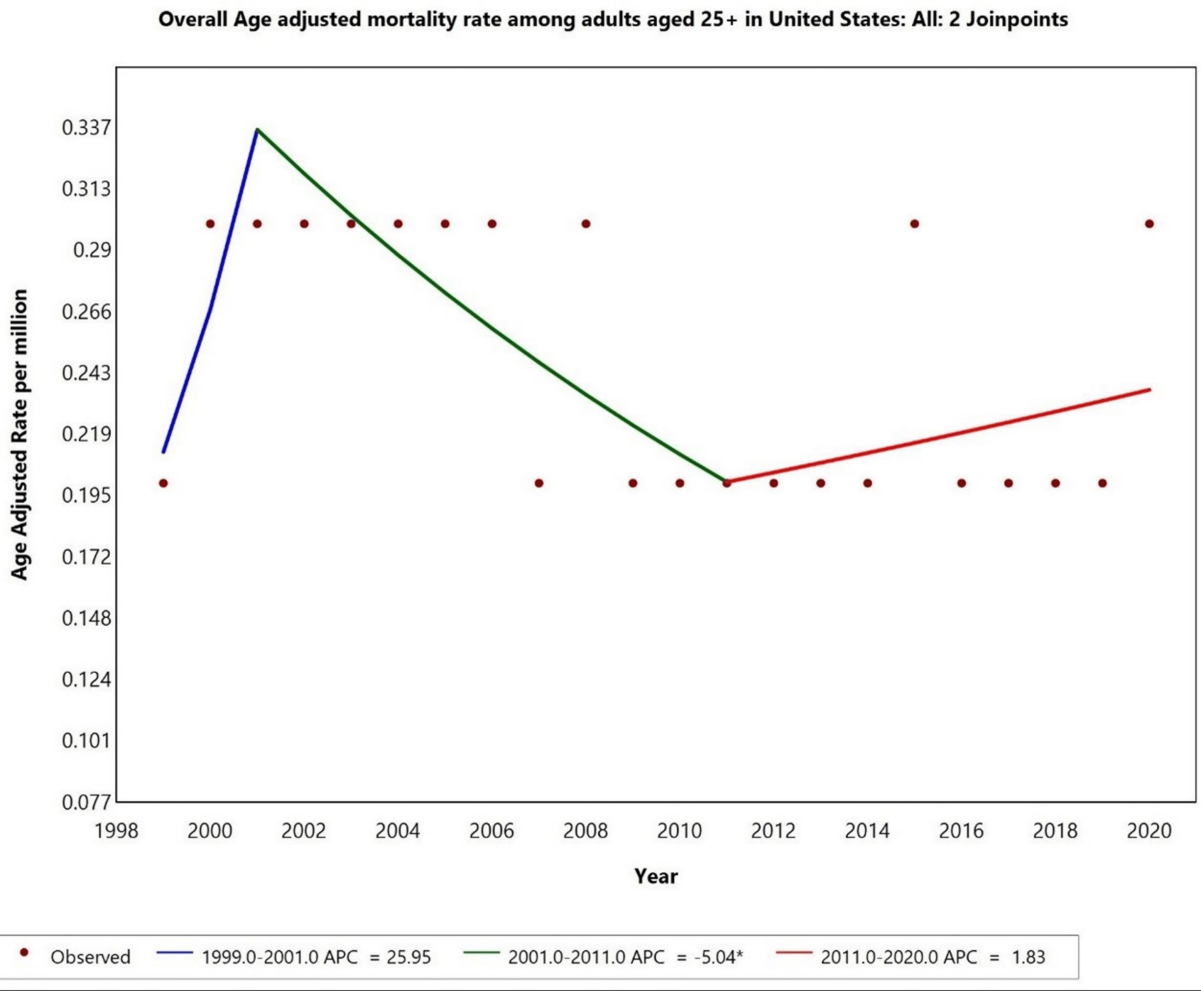
Overall age-adjusted mortality rates among adults aged 25+ in the United States from 1999 to 2020. *Indicates that the annual percentage change (APC) is significantly different from zero at alpha = 0.05 level.

From 1999 to 2020, the AAMR for hyperthyroidism with HF as a contributing cause initially showed an increasing trend from 1999 to 2001, with an annual percentage change (APC) of 25.95% (p < 0.05). A declining trend was noted from 2001 to 2011, with an APC of -5.04% (p < 0.05). However, from 2011 to 2020, the AAMR began to increase, with an APC of 1.83% (p < 0.05). This shift suggests a notable change in mortality patterns over the past two decades, with a recent rise in AAMR (Figure 1).

Gender-specific trends

**Figure 2:**
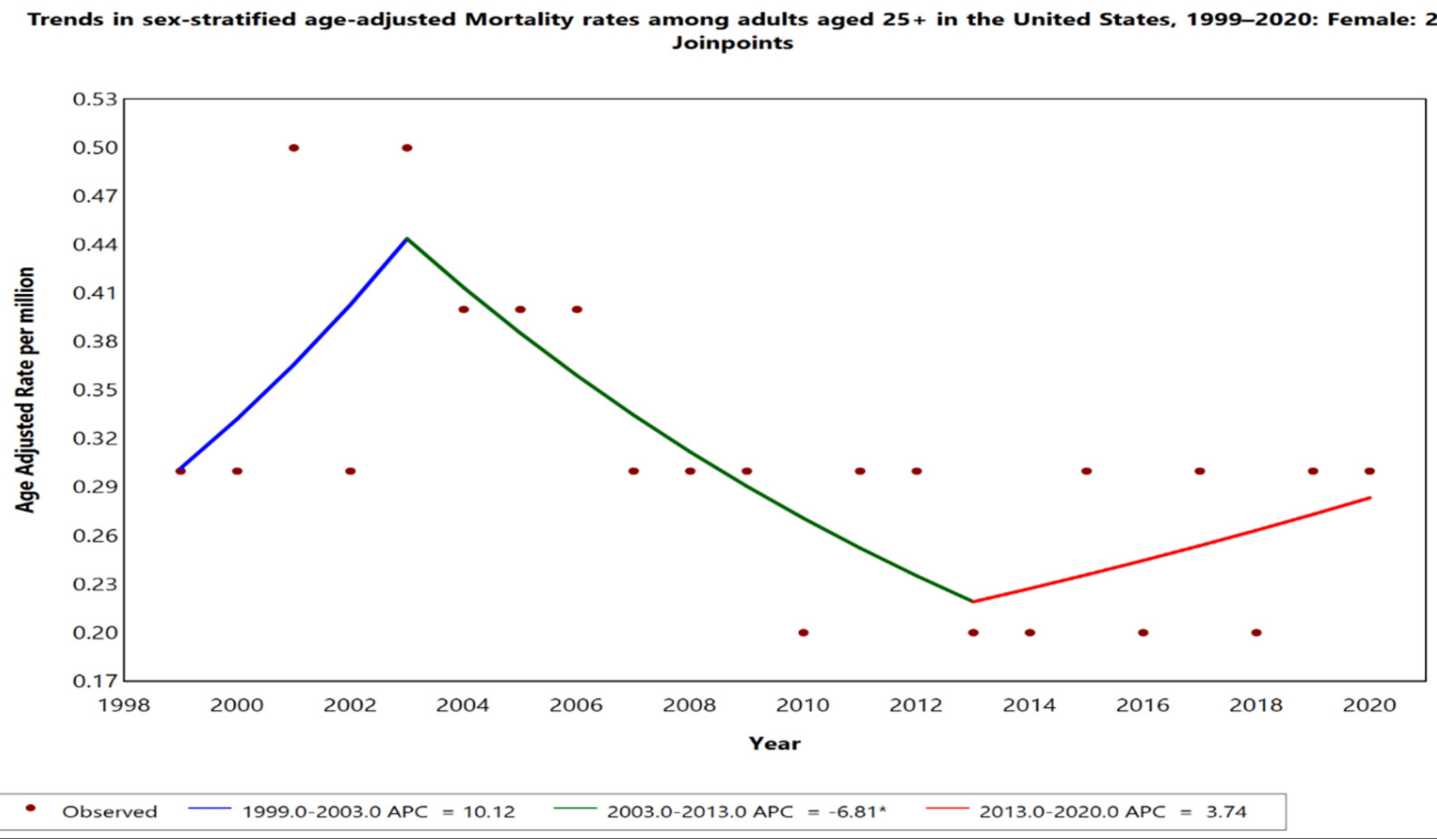
Trends in sex-stratified age-adjusted mortality rates among adults aged 25+ in the United States, 1999–2020. *Indicates that the annual percentage change (APC) is significantly different from zero at alpha = 0.05 level. Temporal trends for the male population are not displayed due to data suppression for counts <10, limiting reliable trend analysis.

When stratified by gender (Figure 2), females showed a fluctuating trend in the AAMR. Between 1999 and 2003, the AAMR increased sharply (+10.12% APC), followed by a significant decline from 2003 to 2013 (APC: -6.81%). Notably, after 2013, the AAMR for females began to rise again (APC: +3.74%), indicating a resurgence in mortality risk in the past decade. Temporal trends for males were not displayed due to data suppression for counts <10, limiting reliable trend analysis.

Race-specific trends

**Figure 3:**
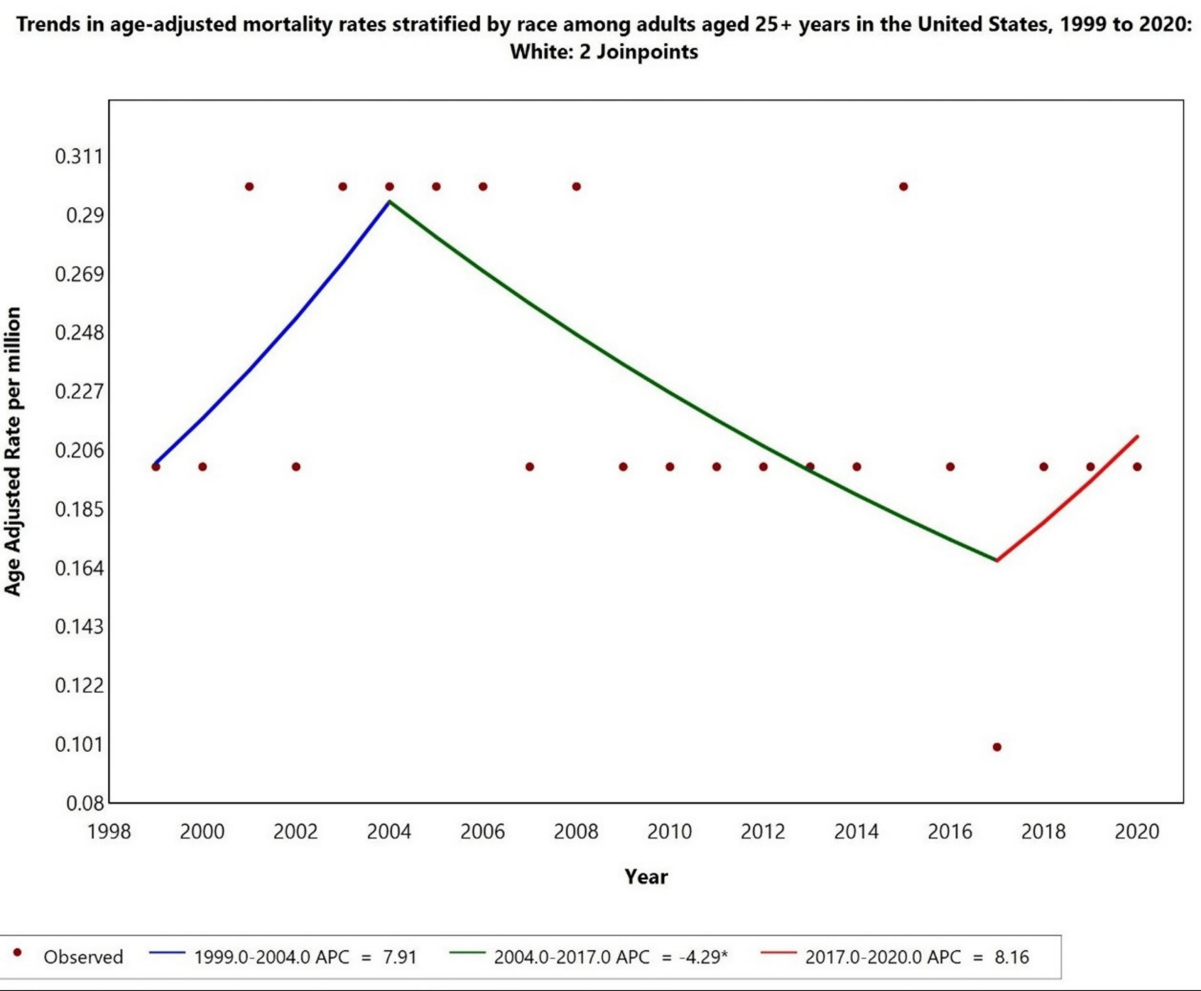
Trends in age-adjusted mortality rates stratified by race among adults aged ≥25 years in the United States, 1999 to 2020. *Indicates that the annual percentage change (APC) is significantly different from zero at alpha = 0.05 level. Temporal trends for American Indian/Alaska Native, Asian Pacific Islander, and Black or African American populations are not displayed due to data suppression for counts <10, limiting reliable trend analysis.

Racial disparities were observed in hyperthyroidism-related mortality, with HF as a contributing factor. White individuals had the highest AAMR, with a significant increase from 1999 to 2004 (APC: +7.91%), followed by a declining trend from 2004 to 2017 (APC -4.49%). However, from 2017 to 2020, the AAMR in white individuals showed an increasing trend in APC (8.16%). Trends for American Indian/Alaska Native, Black or African American, and Asian Pacific Islander populations were not displayed due to data suppression for counts <10, limiting reliable trend analysis (Figure 3).

## Discussion

The present study evaluated temporal trends in mortality due to hyperthyroidism with HF as a contributing cause from 1999 to 2020 in the US, focusing on demographic characteristics such as sex, race, and urbanization levels. A total of 1,189 deaths were recorded during this period, with a crude mortality rate of 0.3 per million. The study population was diverse, including various age groups, genders, races, and urbanization categories. Analysis revealed that mortality was disproportionately higher among females (832; 70%) than among males (357; 30%). In addition, a significant majority of deaths occurred in metropolitan areas (75.4%), with large central metropolitan areas accounting for the largest share (27.5%).

A closer examination of the data indicated that White individuals constituted the majority of deaths (73.93%), followed by Black or African American individuals (22.46%), and Asian or Pacific Islanders represented a smaller proportion (3.03%). Urbanization analysis showed that metropolitan areas, particularly large central metro regions, had higher mortality rates than non-metropolitan areas. The observed sex disparity aligns with prior research, indicating that hyperthyroidism, particularly Graves’ disease, is more prevalent in women, potentially due to autoimmune factors and hormonal influences [16]. Studies have shown that women are more likely to develop thyrotoxicosis, which may exacerbate HF by increasing the heart rate, cardiac output, and myocardial oxygen demand [17]. In addition, underdiagnosis and variability in the treatment of hyperthyroidism in women could contribute to higher mortality rates [18].

The racial disparities observed in this study are consistent with previous findings, indicating higher rates of thyrotoxicosis-related complications in Black individuals than in White and Asian individuals [19]. This may be attributed to differences in healthcare access, socioeconomic status, and underlying comorbidities, such as hypertension and diabetes, which can exacerbate the impact of hyperthyroidism on cardiac function [20]. For instance, Black patients with hyperthyroidism are less likely to receive timely antithyroid treatment, potentially increasing the risk of HF and related mortality [21].

The higher mortality observed in metropolitan areas than in non-metropolitan areas may reflect disparities in healthcare access, environmental stressors, and socioeconomic status. Previous research suggests that individuals in urban settings may face higher stress levels, environmental pollution, and limited access to preventive healthcare services, which could exacerbate the cardiovascular effects of hyperthyroidism [22]. Conversely, the lower mortality in non-metropolitan areas might reflect differences in lifestyle factors, although it could also indicate underreporting or disparities in the healthcare infrastructure.

The association between hyperthyroidism and HF is well documented, with thyrotoxicosis known to induce high-output HF through mechanisms such as increased sympathetic activity, elevated myocardial contractility, and tachycardia [23]. Excess thyroid hormones can lead to atrial fibrillation, increased left ventricular mass, and diastolic dysfunction, further complicating HF outcomes [24]. The observed mortality trends in this study underscore the need for timely diagnosis and management of hyperthyroidism, particularly in high-risk demographic groups, such as the elderly.

These findings underscore the need for targeted public health interventions to improve the screening and management of hyperthyroidism in populations at a higher risk of HF. Expanding access to antithyroid therapies and beta-blockers, particularly in metropolitan areas with high mortality rates, may help mitigate these disparities [25]. Public health campaigns focusing on early diagnosis and treatment adherence, especially among women and racial minorities, can also play a significant role in reducing mortality. The study findings suggest significant associations between demographic factors and mortality due to hyperthyroidism, with HF as a contributing cause; however, the observational nature of the analysis limits causal inference. Future research should explore the impact of treatment modalities such as radioactive iodine therapy and antithyroid drugs on mortality outcomes in diverse populations using longitudinal designs [26]. In addition, studies examining the role of comorbidities, such as atrial fibrillation and hypertension, could provide a more comprehensive understanding of the observed disparities.

Limitations

This study had several limitations. First, the analysis was based on death certificate data, which may introduce classification bias, particularly in cases where hyperthyroidism or HF may not have been accurately recorded as the primary cause of death. The misclassification of hyperthyroid subtypes could also affect the accuracy of our findings. The reliance on ICD codes may not capture the full spectrum of hyperthyroidism presentations, particularly in underrepresented populations. In addition, data suppression for counts less than 10 limited the ability to comprehensively analyze mortality trends for certain subgroups, such as the American Indian or Alaska Native populations.

The cross-sectional design of this study precludes any conclusions regarding causality. While significant associations were observed between demographic factors and mortality, it remains unclear whether these relationships are causal or confounded by unmeasured variables such as socioeconomic status, healthcare access, or underlying comorbidities. The use of aggregated data may also mask regional variations in mortality, which could provide valuable insights into targeted interventions. Furthermore, the absence of information on treatment patterns, medication adherence, and lifestyle factors such as diet and physical activity limits the ability to fully contextualize the observed mortality trends.

Another limitation was the potential urbanization bias. The higher mortality observed in metropolitan areas may reflect better reporting systems and healthcare infrastructure rather than a true difference in mortality risk. The inability to adjust for healthcare access and quality further limits the interpretability of these findings. Finally, the exclusion of data for certain racial groups due to suppression rules restricts the generalizability of the findings to all racial and ethnic groups in the US. Future studies should address these limitations through more robust study designs, including prospective cohort studies, and by incorporating clinical data to validate death certificate information.

## Conclusions

This study highlights the rising mortality trends in patients with hyperthyroidism and HF, with the highest burden in White individuals, females, and metropolitan regions. The AAMR initially showed an increasing trend from 1999 to 2001, followed by a declining trend from 2001 to 2011, and then again, an increasing trend from 2011 to 2010, particularly among females and White individuals, showing evolving disparities in the trends. These findings emphasize the need for improved healthcare access to address racial and gender disparities. Future research should explore early risk assessment and better prevention strategies for early diagnosis to help improve the long-term outcomes of patients.

## References

[1] Kravets I (2016). Hyperthyroidism: diagnosis and treatment. Am Fam Physician.

[2] Reid JR, Wheeler SF (2005). Hyperthyroidism: diagnosis and treatment. Am Fam Physician.

[3] McLeod DS, Cooper DS, Ladenson PW, Whiteman DC, Jordan SJ (2015). Race/ethnicity and the prevalence of thyrotoxicosis in young Americans. Thyroid.

[4] Inman BL, Long B (2023). Thyrotoxicosis. Emerg Med Clin North Am.

[5] Xu T, Zheng X, Wei T (2023). Preoperative preparation for Graves' disease. Front Endocrinol (Lausanne).

[6] Bozkurt B, Ahmad T, Alexander K (2025). HF STATS 2024: heart failure epidemiology and outcomes statistics an updated 2024 report from the Heart Failure Society of America. J Card Fail.

[7] Mehta PA, Cowie MR (2006). Gender and heart failure: a population perspective. Heart.

[8] Yu B, Akushevich I, Yashkin AP, Yashin AI, Lyerly HK, Kravchenko J (2022). Epidemiology of geographic disparities in heart failure among US older adults: a Medicare-based analysis. BMC Public Health.

[9] Martin SS, Aday AW, Allen NB (2025). 2025 heart disease and stroke statistics: a report of US and global data from the American Heart Association. Circulation.

[10] Razvi S, Jabbar A, Pingitore A (2018). Thyroid hormones and cardiovascular function and diseases. J Am Coll Cardiol.

[11] Ertek S, Cicero AF (2013). Hyperthyroidism and cardiovascular complications: a narrative review on the basis of pathophysiology. Arch Med Sci.

[12] Udani K, Patel D, Hart L, Nambudiri V (2021). Impact of hyperthyroidism on in-hospital outcomes of patients with heart failure. J Community Hosp Intern Med Perspect.

[13] Fan SW, Ong LT (2024). Prevalence and risk factors of heart failure in patients diagnosed with hyperthyroidism: a systematic review and meta-analysis. touchREV Endocrinol.

[14] (2025). Multiple cause of death data on CDC WONDER. https://wonder.cdc.gov/mcd.html.

[15] (2025). HIPAA privacy rule and its impacts on research. https://privacyruleandresearch.nih.gov/.

[16] Kahaly GJ, Grebe SK, Lupo MA, McDonald N, Sipos JA (2011). Graves' disease: diagnostic and therapeutic challenges (multimedia activity). Am J Med.

[17] Klein I, Ojamaa K (2001). Thyroid hormone and the cardiovascular system. N Engl J Med.

[18] Biondi B, Cooper DS (2008). The clinical significance of subclinical thyroid dysfunction. Endocr Rev.

[19] Hollowell JG, Staehling NW, Flanders WD, Hannon WH, Gunter EW, Spencer CA, Braverman LE (2002). Serum TSH, T(4), and thyroid antibodies in the United States population (1988 to 1994): National Health and Nutrition Examination Survey (NHANES III). J Clin Endocrinol Metab.

[20] Cappola AR, Fried LP, Arnold AM (2006). Thyroid status, cardiovascular risk, and mortality in older adults. JAMA.

[21] Gillis A, Chen H, Wang TS, Dream S (2024). Racial and ethnic disparities in the diagnosis and treatment of thyroid disease. J Clin Endocrinol Metab.

[22] Mensah GA, Mokdad AH, Ford ES, Greenlund KJ, Croft JB (2005). State of disparities in cardiovascular health in the United States. Circulation.

[23] Fazio S, Palmieri EA, Lombardi G, Biondi B (2004). Effects of thyroid hormone on the cardiovascular system. Recent Prog Horm Res.

[24] Khan R, Sikanderkhel S, Gui J (2020). Thyroid and cardiovascular disease: a focused review on the impact of hyperthyroidism in heart failure. Cardiol Res.

[25] Matsuo Y, Jo T, Watanabe H, Matsui H, Fushimi K, Yasunaga H (2024). Clinical efficacy of beta-1 selective beta-blockers versus propranolol in patients with thyroid storm: a retrospective cohort study. Crit Care Med.

[26] Liu X, Wong CK, Chan WW, Tang EH, Woo YC, Lam CL, Lang BH (2021). Outcomes of Graves' disease patients following antithyroid drugs, radioactive iodine, or thyroidectomy as the first-line treatment. Ann Surg.

